# [Corrigendum] 2'-Hydroxyflavanone inhibits epithelial-mesenchymal transition, and cell migration and invasion via suppression of the Wnt/β-catenin signaling pathway in prostate cancer

**DOI:** 10.3892/or.2025.8935

**Published:** 2025-06-26

**Authors:** Shiqi Wu, Jun Huang, Ke Hui, Yangyang Yue, Yanan Gu, Zhongyun Ning, Xinyang Wang, Dalin He, Kaijie Wu

Oncol Rep 40: 2836–2843, 2018; DOI: 10.3892/or.2018.6678

Following the publication of the above article, the authors contacted the Editorial Office to explain that they had made inadvertent errors in compiling the scratch-wound assay images in Fig. 1B on p. 2838 and Fig. 4A on p. 2840; essentially, the same data had been included for the ‘2HF, 10 µM, 0 h’ and ‘2HF, 5 µM, 12 h’ experiments in Fig. 1B, and for the ‘Vector, 2HF, 5 µM, 12 h’ and ‘2HF, 5 µM, 24 h’ experiments in Fig. 4A.

However, the authors were able to re-examine their original data, and realized how these errors had occurred. The revised and corrected versions of Figs. 1 and 4, now including replacement data for the experiments with the PC-3 cell line (lower panel) in Fig. 1B and the Vector experiments with the DU145 cell line in Fig. 4A, are shown on the next two pages. Note that the errors made with the assembly of the data in these figures did not affect the overall conclusions reported in the paper. The authors apologize to the Editor of *Oncology Reports* and to the readership for any inconvenience caused.

## Figures and Tables

**Figure 1.**
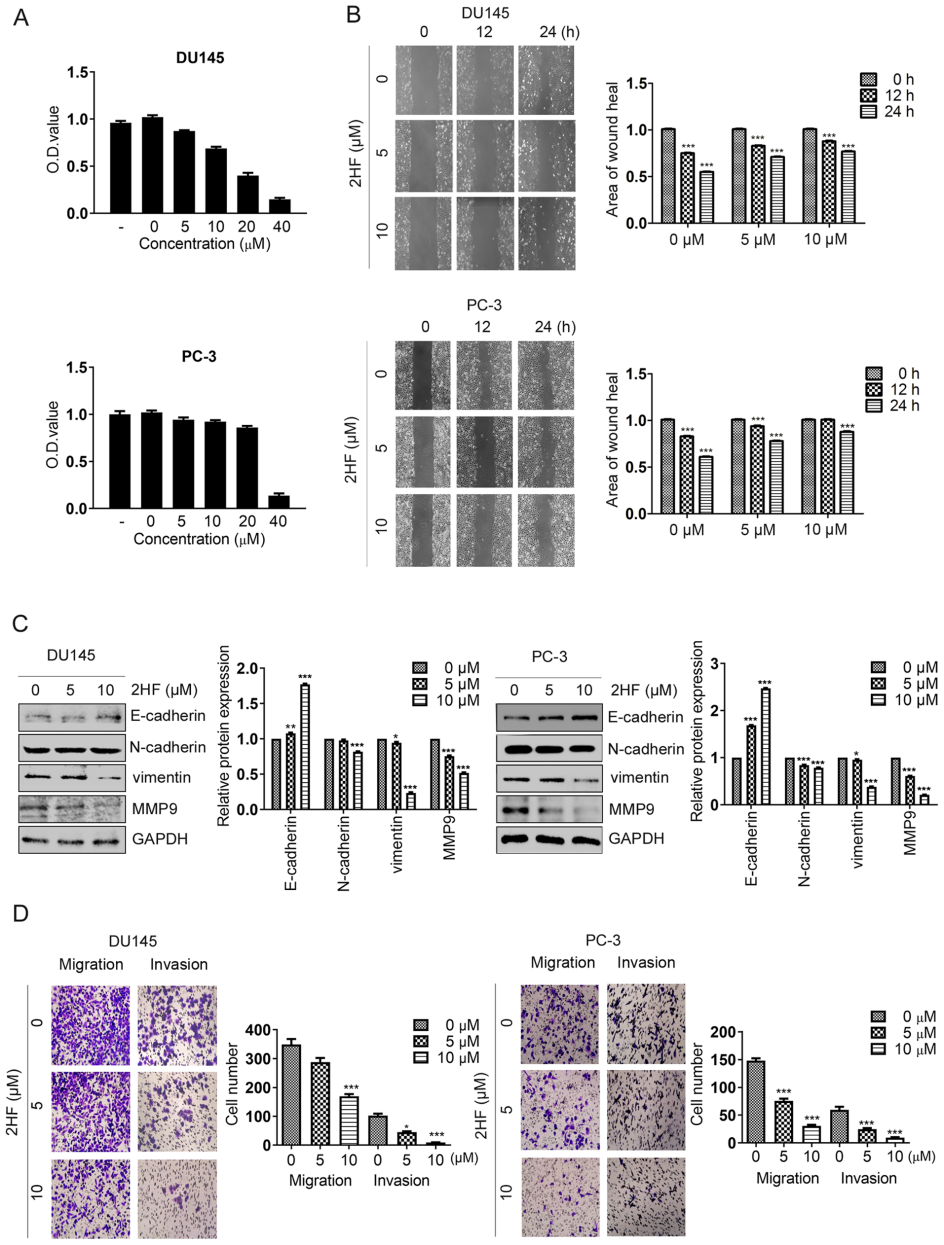
2HF inhibit epithelial-mesenchymal transition, and cell migration and invasion. (A) DU145 and PC-3 cells were treated with different concentrations of 2HF (0, 5, 10, 20 and 40 μM) for 48 h, prior to being subjected to an MTT assay to measure its cytotoxicity to cells. *P<0.05, ***P<0.001. (b) Representative pictures demonstrating the wound healing rate of DU145 and PC-3 cells treated with different concentrations of 2HF. Quantification analysis is shown. ***P<0.001. (C) E-cadherin, N-cadherin, Vimentin and MMP9 proteins were detected by western blot analysis in DU145 and PC-3 cells treated with different concentrations of 2HF. GAPDH was used as a loading control and all the experiments were repeated 3 times. Quantification analysis is shown. *P<0.05, **P<0.01, ***P<0.001 vs. control. (D) Representative pictures of Transwell migration and invasion assays demonstrating the migration and invasion abilities of DU145 and PC-3 cells treated with different concentrations of 2HF. Quantification analysis is shown. *P<0.05, ***P<0.001 vs. control. OD, optical density; MMP9, matrix metalloproteinase 9.

**Figure 4.**
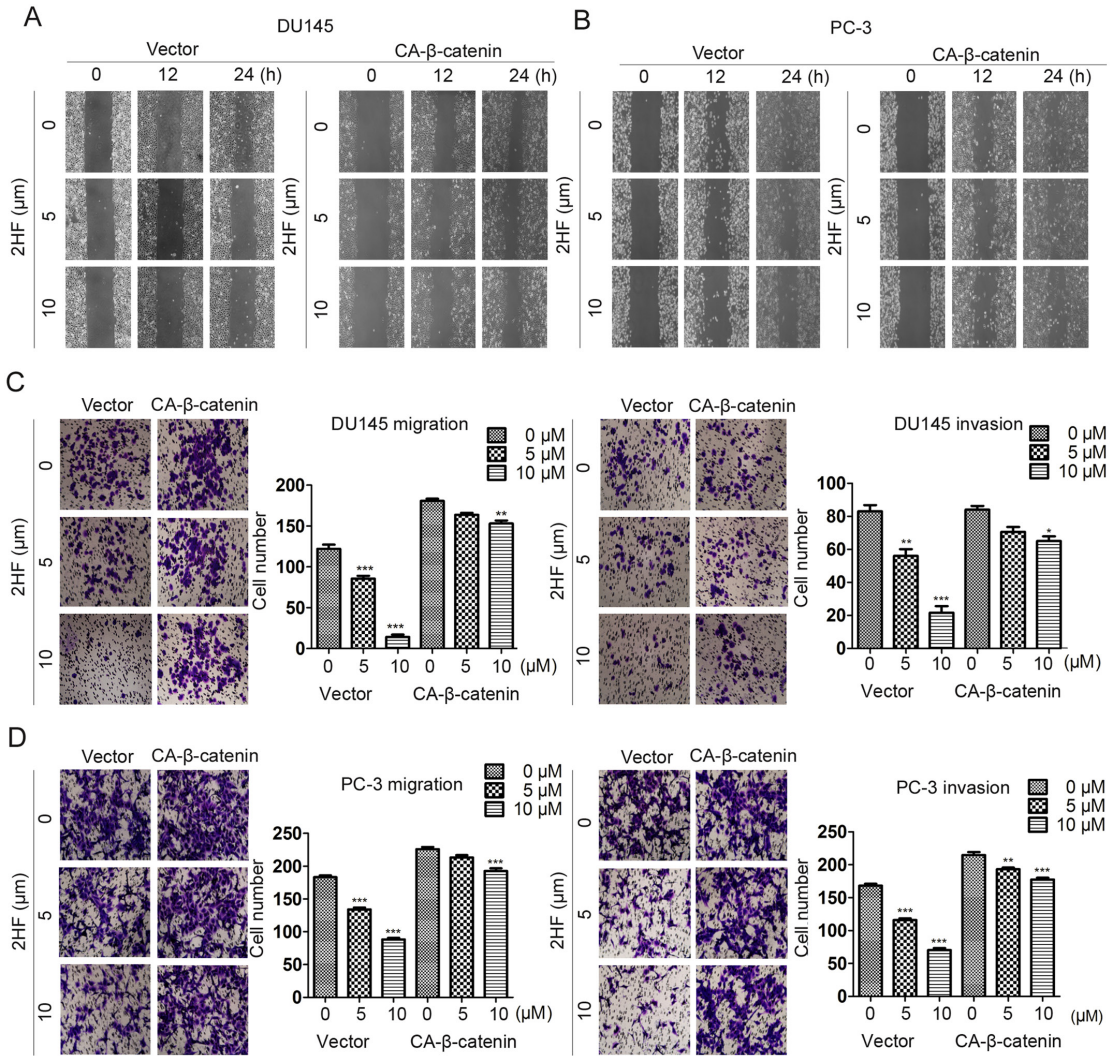
β-catenin abolishes the biological function of 2HF in prostate cancer migration and invasion. Representative images showing the wound healing rate of (A) DU145 and (b) PC-3 cells transfected with CA-β-catenin or control vector and then treated with different concentrations of 2HF. Representative images of Transwell migration and invasion assays showing the migration and invasion abilities of the (C) DU145 and (D) PC-3 cells transfected with CA-β-catenin or control vector and then treated with different concentrations of 2HF. Quantification analysis of Transwell migration and invasion assays is shown. *P<0.05, **P<0.01, ***P<0.001 vs. control.

